# Non-Thermal Electromagnetic Radiation Damage to Lens Epithelium

**DOI:** 10.2174/1874364100802010102

**Published:** 2008-05-21

**Authors:** Elvira Bormusov, Usha P.Andley, Naomi Sharon, Levi Schächter, Assaf Lahav, Ahuva Dovrat

**Affiliations:** 1 Rappaport Faculty of Medicine, Technion – Israel Institute of Technology, Haifa 31096, Israel; 2 Department of Electrical Engineering, Technion – Israel Institute of Technology, Haifa 32000, Israel; 3 Department of Ophthalmology and Visual Sciences, Washington University School of Medicine, St. Louis, Missouri 63110, USA

## Abstract

High frequency microwave electromagnetic radiation from mobile phones and other modern devices has the potential to damage eye tissues, but its effect on the lens epithelium is unknown at present. The objective of this study was to investigate the non-thermal effects of high frequency microwave electromagnetic radiation (1.1GHz, 2.22 mW) on the eye lens epithelium in situ. Bovine lenses were incubated in organ culture at 35°C for 10-15 days. A novel computer-controlled microwave source was used to investigate the effects of microwave radiation on the lenses. 58 lenses were used in this study. The lenses were divided into four groups: (1) Control lenses incubated in organ culture for 10 to15 days. (2) Electromagnetic radiation exposure group treated with 1.1 GHz, 2.22 mW microwave radiation for 90 cycles of 50 minutes irradiation followed by 10 minutes pause and cultured up to 10 days. (3) Electromagnetic radiation exposure group treated as group 2 with 192 cycles of radiation and cultured for 15 days. (4) Lenses exposed to 39.5ºC for 2 hours 3 times with 24 hours interval after each treatment beginning on the second day of the culture and cultured for 11 days. During the culture period, lens optical quality was followed daily by a computer-operated scanning laser beam. At the end of the culture period, control and treated lenses were analyzed morphologically and by assessment of the lens epithelial ATPase activity. Exposure to 1.1 GHz, 2.22 mW microwaves caused a reversible decrease in lens optical quality accompanied by irreversible morphological and biochemical damage to the lens epithelial cell layer. The effect of the electromagnetic radiation on the lens epithelium was remarkably different from those of conductive heat. The results of this investigation showed that electromagnetic fields from microwave radiation have a negative impact on the eye lens. The lens damage by electromagnetic fields was distinctly different from that caused by conductive heat.

## INTRODUCTION

Environmental stress, including electromagnetic radiation, has a negative impact on the lens and is considered a risk factor for cataracts [1,2]. Microwaves from modern technological devices such as cellular phone transmitters and receivers, radars, radio and TV transmitters and video display terminals are an important part of modern life [3,4]. While thermal effects of microwaves have been well characterized and guidelines for exposure to microwave radiation are clear, their non-thermal effects on eye tissues are not known [3]. Cataracts form when proteins in the lens begin to clump and scatter light, and can be induced at high temperature with conductive heat. According to the World Health organization, cataracts are the leading cause of vision impairment worldwide [5,6]. The ocular lens is exposed to environmental stress throughout the lifetime of an individual [7,8]. However, currently very little information is available on the effect of electromagnetic fields from high frequency microwave radiation. With the advent of cellular phones and other devices emitting high frequency electromagnetic radiation, there is a strong rationale for determining the damaging effect of electromagnetic fields generated from high frequency microwave radiation on the eye lens. It has also been recognized that a particularly vulnerable group might be children, as they are likely to have the highest cumulative exposure to radiowaves from mobile devices [9].

The ocular lens is a unique tissue with a distinctive cellular architecture. The lens consists of two cell types, the single layer of lens epithelial cells on its anterior surface, which are responsible for the growth and development of the entire lens, and the differentiated lens fiber cells, with elongated morphology that do not turn over their proteins throughout life. Lens epithelial cells provide metabolic support to the entire lens, and are also the first cells in the lens to be exposed to damaging radiation [10,11]. Moreover, the epithelium is the site at which metabolic enzymes and transport systems are concentrated, thus making these cells essential for maintaining lens homeostasis, and as the first line of defense against environmental damage [12].

Heat stress delivered by microwaves to an organism unavoidably entails exposure of the cells to oscillating electrical and magnetic fields, raising the possibility that non-thermal, direct electromagnetic field-mediated effects could cause different effects on cells than the effects of stress delivered by heat conducted from the environment. Indeed, it has been shown that microwaves (2.45 GHz) cause a significantly higher degree of protein unfolding than conventional heating for protein solutions [4]. High frequency electromagnetic radiation effects of microwaves have been investigated in several cell culture systems. Human A172 cells exposed to 2.45 GHz demonstrate a transient increase in heat shock protein Hsp27 phosphorylation at very high SAR (>100W/kg) [13]. In contrast, microarray analysis of gene expression in a human glioblastoma cell line exposed to 1.9 GHz radiofrequency field showed no effect on gene expression [14]. Similarly, murine m5S cells exposed to 2.45 GHz for 2 hours up to specific absorption ratios (SAR) of 100 W/kg do not show chromosomal aberrations [15]. However, studies on cultured lens epithelial cells suggest changes in protein expression with an up-regulation of heat shock protein 70 upon exposure to microwaves [16].

Lens opacity can be determined *in vitro* by a sensitive optical monitoring method developed for organ cultured lenses [17]. Previously, our laboratory developed a novel experimental system to investigate environmental effects on the intact eye lens [18,19]. We used this system to study the non-thermal effects of high frequency electromagnetic fields on the eye lens. In the present paper we demonstrate damage to lens epithelium by non-thermal effects and demonstrated that these changes are distinct from thermal effects. Our results further show that while damage to lens optical quality is reversible, microwaves induce irreversible damage to lens epithelial cells.

## MATERIALS AND METHODOLOGY

### Lenses and Organ Culture System: 

Eyes were obtained from 1-year-old male calves at an abattoir. Lenses were dissected within 2-4 hours after enucleation under sterile conditions. Each lens was placed in a glass and silicon rubber chamber containing 24 ml culture medium (M199 with Earl’s balanced salt solution, supplemented with 5.96 g/l HEPES, 3% dialyzed fetal calf serum and the antibiotics penicillin 100 U/ml and streptomycin 0.1 mg/ml). The glass chamber was filled with sufficient culture medium to completely immerse the lenses. The lenses were incubated at 35°C. Culture medium was changed daily. Experimental treatments began after pre-incubation of 24 hours. Damaged lenses were excluded prior to experimental treatment [19].

### Experimental treatment: 

Bovine lenses were incubated in organ culture conditions for 10 to 15 days. The lenses were divided into four groups:

1. Control group (20 lenses) was untreated and kept in culture for 10 to 15 days.
2. Electromagnetic radiation exposure group (12 lenses) receiving microwaves at 1.1GHz, 2.22 mW for 90 cycles of 50 minutes each followed by 10 minutes pause and kept in culture up to 10 days.
3. Electromagnetic radiation exposure group (20 lenses) receiving microwaves at 1.1GHz, 2.22mW for 192 cycles of 50 minutes each followed by 10 minutes pause and kept in culture up to 15 days.
4. Conductive heat treatment group (6 lenses) exposed to 39.5ºC for 2 hours three times, with 24 hours intervals beginning on the second day of the culture, and incubated in culture for 11 days.

During culture lens optical quality was followed daily. At the end of the culture period control and treated lenses were taken for morphological analysis by inverted microscopy and for staining of lens epithelial layer for ATPase activity.

### Electromagnetic System: 

A computer-controlled microwave source was built to feed four transmission lines surrounding each culture chamber containing a single lens. It consists of a voltage-controlled oscillator (VCO) generating a constant microwave power (13 dBm). Since it is necessary to control the radiation intensity the lenses are being exposed to, the output from the VCO is attenuated by two attenuators: one fixed and the other variable (the latter being computer-controlled), permitting the desired degree of freedom regarding the exposing intensity. In order to restore the power levels to the desired intensities, the microwave signal is directed into a 30 dB amplifier which has a maximum output of 1 W before saturation occurs. A four arm power-splitter provides each transmission line with a microwave signal attenuated by 7 dB relative to the output of the amplifier. The signal traversing the transmission line is absorbed by a matched termination. The detailed calibration process has been described previously [18]. For experimental treatment of lenses, two different exposure modes of 1.1GHz, 2.22mW microwave radiation were used. At the higher dose lenses were exposed to 192 cycles each of 50 minute exposure, followed by a 10 minute pause. The exposure was continuous from day 2 to day 9 of culture. The lower dose was of 90 cycles that also began on day 2 but ended on day 6 of the culture.

### Optical Monitoring System: 

An optical bench [20] was used for daily testing of both exposed and control lenses. A 670 nm diode laser with the beam parallel to the axis of the lens was directed towards the cultured lens along one meridian in 0.5 mm increments. After passing through the lens, the laser beam is refracted and the system determines the back vertex focal length for every beam position. Each scan consists of measurements of the same beam from 22 different points across the lens. This optical monitoring apparatus uses a computer-operated scanning laser beam, a video-camera system and a video frame analyzer to record the focal length and transmittance of the cultured lens. The scanner is designed to measure the focal length at points across the diameter of the lens. The lens container permits the lens to be exposed to a vertical laser beam from below. The laser source projects its light onto a plain mirror, which is mounted at 45° on a carriage assembly. The mirror reflects the laser beam directly up through the test lens. The mirror carriage is connected to a positioning motor, which moves the laser beam across the lens. The camera sees the cross section of the beams and, by examining the image at each position of the mirror, Scan-Tox software is able to measure the quality of the lens by calculating the back vertex distance for each beam position.

A lens of good optical quality is able to focus the laser beam from the various locations at a fairly sharp focal point [19,20]. When the lens is damaged, it fails to intersect these laser beams at a consistent focal point. Measurements of variability of focal length is a quantitative measure of lens damage.

### Lens Epithelium Microscopy and Histochemistry: 

Microscopic changes in the lenses exposed to microwaves or conductive heat treatment were analyzed by photographing the lenses in an inverted microscope. After 10 to 15 days in culture lenses were photographed. In addition, the lens capsule was carefully removed, and the epithelium was analyzed for ATPase activity. Histochemical analysis of magnesium-activated Na,K ATPase was performed according to [21].

## RESULTS

Fig. (**1**) demonstrates the optical quality of control lenses and lenses exposed to microwaves. For each time of microwave exposure, damage reached a maximum approximately four days after beginning of exposure. At the shorter time of exposure (90 cycles) lens damage began to decline as soon as the microwave exposure stopped on day 6 of the culture. However, damage continued in lenses receiving 192 cycles of microwave exposure, with the variation in focal length remaining high up to the time when the radiation exposure ended on day 9 of culture. Our optical measurements clearly demonstrate that the lens begins to recover from microwave damage, which is demonstrated by the reduced focal length variability measurements. Lenses were analyzed microscopically at the end of the analysis of focal length variability (days 10 to 15 of culture).

**Fig. (2).**
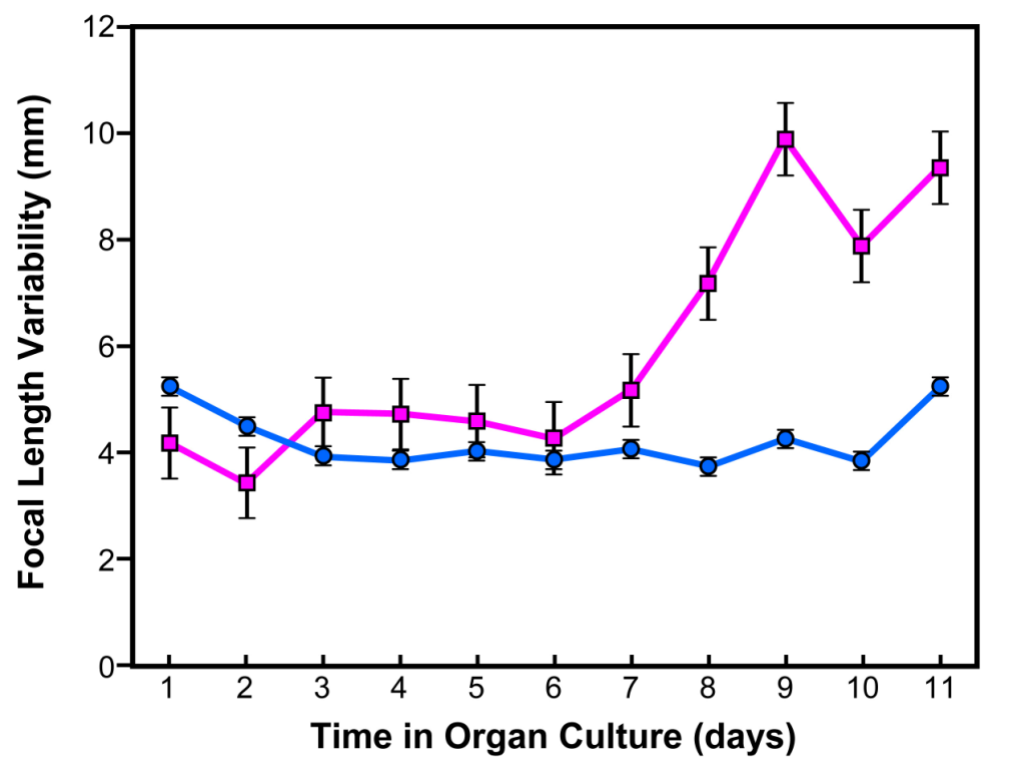
Focal length variability measurements in bovine lenses exposed to conductive heat stress. The lenses were exposed to 39.5°C for 2 hours on days 2, 3 and 4 of culture. Note that lens damage appeared from day 7 of the culture and there was no recovery. Control lenses are shown by the *blue line* and heat-treated lenses by the *pink line*.

To compare the effects of microwaves with those of conductive heat, lenses were warmed to 39.5ºC (Fig. **2**). Lenses were exposed to two hours of heat three times with 24 hours interval between treatments beginning on day 2 of the culture. The lenses were kept in culture for 11 days. These results demonstrate the optical quality of lenses exposed to heat.

Fig. (**3**) demonstrates inverted microscope photographs of control and treated lenses. Lenses exposed to electromagnetic treatment of 90 cycles show almost no damage when the lens is viewed at low magnification, but at high magnification it is clear that damage is located at the lens epithelial layer.

Lenses exposed to 192 cycles demonstrate damage to lens epithelium at both magnifications. The cell layer was not intact, there were damaged cells demonstrated by the black spots. Lenses exposed three times to a temperature of 39.5ºC for 2 hours showed a profoundly different pattern of damage. The damage appeared as bubbles on the surface and between the cells. The appearance of lens epithelial cells in Fig. (**3F**) (exposure to 192 cycles of microwave radiation) and Fig. (**3H**) (exposure to heat) clearly demonstrates the difference between electromagnetic radiation and heat.

A series of temperature measurements in the vicinity of the lens, inside the vessel, in the tissue culture fluid were performed during exposure to the microwave radiation, and the temperature was found to be stable and consistent with the temperature monitored by the incubator probe (35ºC).

**Fig. (4).**
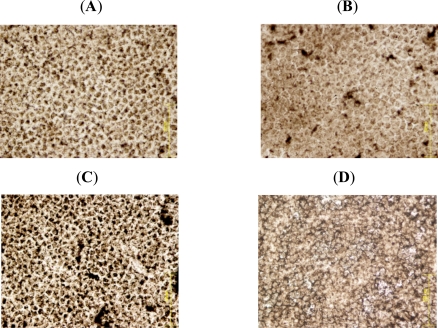
Staining for lens epithelial ATPase activity. **(A)** Control lens epithelium shows a homogenous cell layer with brown spots of ATPase activity at the cell membrane. **(B)** Exposure to 90 cycles of microwave electromagnetic radiation caused some changes in the cells, there are spots of increased ATPase activity and the cells are larger than control cells. **(C)** 192 cycles of electromagnetic radiation caused dramatic increase in ATPase activity, with abnormal cell morphology and dead cells with no ATPase activity. **(D)** Conductive heat-treated lenses. Treatment of lenses at 39.5^°^C caused the epithelial cells to swell. ATPase activity could not be detected. Scattered between the swollen cells were small cells with high ATPase activity. Comparison between **(C)** and **(D)** demonstrates that the effects of micorwaves on the lens epithelium are remarkably different from the effects of heat.

Lens capsule-epithelium was removed and flat mounts were prepared. Staining the cells for ATPase activity shows homogenous cells with uniform brown spots of ATPase activity at the cell membrane in the control lens epithelium (Fig. **4A**). Exposure to 90 cycles of microwaves caused some changes in the cells, with spots of increased ATPase activity and an increase in cell size (Fig. **4B**). 192 cycles of microwave exposure caused a dramatic increase in ATPase activity, accompanied by a loss of normal cell appearance and damaged cells with no ATPase activity (Fig. **4C**). Heating to 39.5ºC caused the cells to swell and lose their ATPase activity (Fig. **4D**). Interestingly, scattered between the swollen cells were small cells with high ATPase activity. The differences between Fig. (**4C**) and (**4D**) demonstrate that the effects of microwaves on the lens epithelium are remarkably different from the effects of conductive heat.

## DISCUSSION

Environmental stress, including electromagnetic radiation, has a negative impact on the lens and is considered a risk factor for cataracts [3,6]. In this study we show non-thermal effects of microwaves at the same frequency that is used in cellular phones on the intact lens in organ culture. Microwaves damaged the lens optical quality, as measured by focal length variability. Microwave damage was dose-dependent and at the doses tested was reversible when exposure stopped. Lens morphology was strikingly different in microwave-exposed and conductive heat-exposed lenses, indicating that the effects due to microwaves are different from thermal effects. Remarkably, microwave damage was seen in each of the parameter analyzed. Our results demonstrate that while microwave damage to optical quality was reversed when the exposure was stopped, morphological changes to the epithelium were irreversible.

The doses we used are similar to those the lens receives when we speak on a cell phone; however, cell phone use is never continuous for a period of 8 days and nights. On the other hand, the damage by intermittent use of cell phones can be cumulative. It is also important to consider that the lens *in vivo* likely has better repair mechanisms than the lens under the culture conditions used in our study. The damage that we are seeing in culture conditions after 15 days of exposure is likely to appear *in vivo* much later- even 10 or 20 years later. It is recommended to use cell phones from a distance to minimize exposure, thus reducing any potential harmful effects of cell phone use on the lens.

Previous studies suggest that the effect of microwaves on protein conformation and function is not the same as the effects of ambient heating with conducted heat at the same temperature [22-24]. Microwaves exerted a greater effect on proteins than could be explained on the basis of temperature changes alone. Similarly, expression of heat shock protein 70 and phosphorylation of HSP27 were not consistent with the effects of temperature rise alone [13]. In our study, we found that conductive heat altered the optical quality of the lens but in contrast to microwave exposure, damage to lens optical quality by conductive heat was not repaired under the time frame of these experiments. Moreover, the nature of change with conductive heat on the lens epithelium was very distinct from microwave exposure. The mechanism of non-thermal effects on lens epithelium is unknown at present. It has been suggested that the enhanced unfolding of proteins during microwave exposure results from resonant absorption of electromagnetic energy to higher vibration states of the protein or its bound water envelope, causing these vibrational states to be out of thermal equilibrium with the remaining vibrational states [4]. Oscillating electrical fields may also cause unwinding of peripheral regions of protein backbone thereby promoting unfolding [25]. Another possibility is the absorption of high frequency microwaves by bound water [26]. Based on the results of our study, further work is warranted in order to understand the mechanism of damage to the lens epithelium by high frequency microwaves.

In summary, our study shows the damaging effects of high frequency microwave radiation on the intact lens and demonstrates that the optical damage can be recovered. However, the data suggest that more exposures to microwave can cause cumulative damage and can cause cataract.

## Figures and Tables

**Fig. (1).**
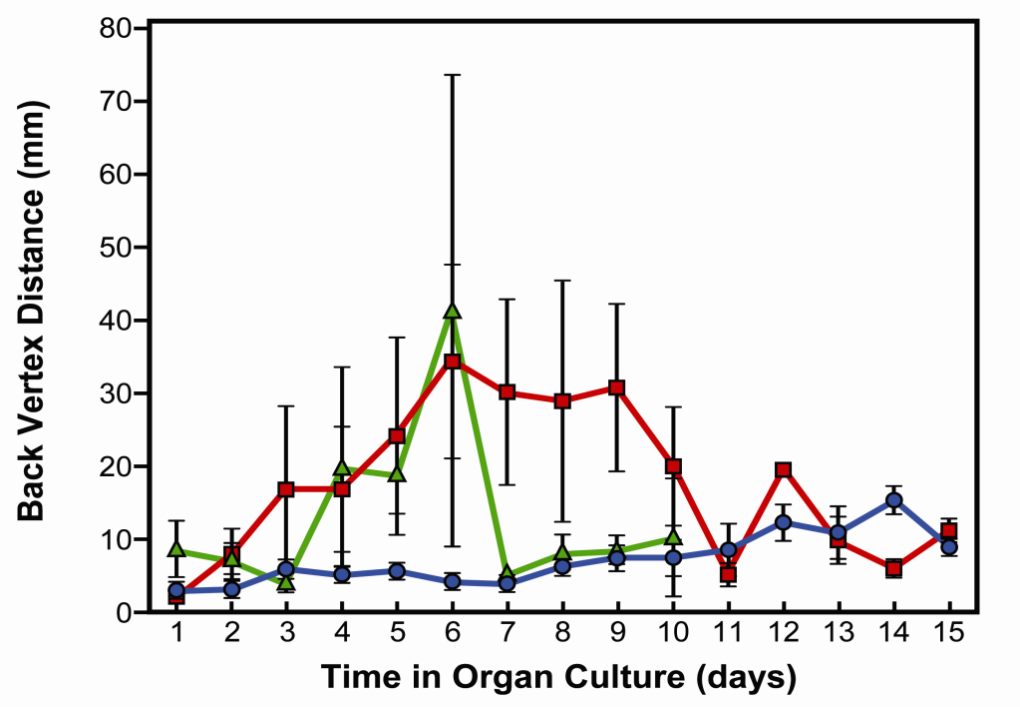
Focal length variability measurements in bovine lenses exposed to high frequency microwaves. Experimental treatments were initiated after pre-incubation of 24 hours. Only non-damaged lenses were included in the study. Control lenses were incubated in culture conditions for 15 days. Treated lenses were exposed 90 or 192 cycles of 50 minutes irradiation at 1.1 GHz and 2.22 mW, followed by 10 minutes pause start after 24 hours pre-incubation. The 90 cycles ends on day 6 of the culture and the 192 cycles on day 10 of the culture period. Focal length variability measurements show a change in lens optical quality by irradiation. The optical quality of the lenses was determined by measuring the focal length at different points for each lens. The focal length variability represents the variation in the 22 focal length measurements during each scan. The focal length variability is measured as the standard error (SE) of the focal length. Note that control lenses (*blue line*) show almost no variation in focal length during the 15 days of culture. However, lenses exposed to 90 (*green line*) or 192 cycles (*red line*) of microwave showed an increase in focal length variability two days after microwave exposure, and then returned to normal after the exposure was stopped.

**Fig. (3).**
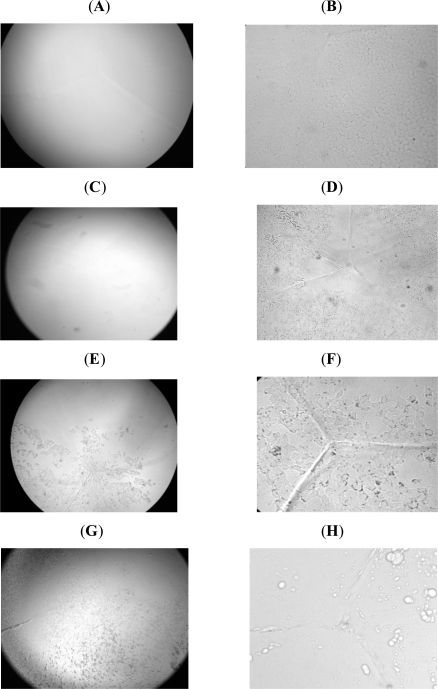
Cytology changes of lens epithelial cells were observed after exposure to microwave electromagnetic radiation or conductive heat. **(A,B)** Control lenses at two different magnifications **(A)** 25X and **(B)** 100X showed clear lenses with an intact lens epithelial layer at both magnifications. **(C)** Lenses exposed to microwaves (90 cycles) showed almost no damage at low magnification. **(D)** At high magnification damage was clearly located at the lens epithelial layer. **(E,F)** Lenses exposed to 192 cycles demonstrated damage to the lens epithelium at both magnifications. The cell's layer was not intact, cells were missing as demonstrated by the black spots. Note the prominence of the suture and enhancement of cell death in the microwave exposed lenses. **(G,H)** Lenses exposed to conductive heat at 39.5^°^C showed bubbles of damaged cells. Comparison between **F** and **H** showed a remarkably different lens morphlogical change by microwaves and conductive heat.
